# The optimal and safe intensity for facial nerve stimulation during intraoperative neuromonitoring in middle ear surgery

**DOI:** 10.1371/journal.pone.0221748

**Published:** 2019-08-29

**Authors:** Euyhyun Park, Hyunjung Kim, Hye Min Han, In Hak Choi, Hak Hyun Jung, Gi Jung Im

**Affiliations:** Department of Otolaryngology-Head and Neck Surgery, Korea University College of Medicine, Seoul, Republic of Korea; Kaplan Medical Center, ISRAEL

## Abstract

**Objective:**

This study aimed to investigate the optimal and safe intensity for facial nerve stimulation during middle ear surgery.

**Methods:**

Thirty-seven patients who had their facial nerve exposed prior to surgery were prospectively enrolled in this study, and electromyography (EMG) recordings were obtained from the orbicularis oculi and orbicularis oris muscles. Four pigs were also enrolled in an animal study, and continuous stimulation was performed on the facial nerves of the pigs for 10 minutes. The EMG responses were measured and the pathologic outcomes of the facial nerve after stimulation were determined.

**Results:**

In the human study, the mean intensity of the minimal electrical stimulation threshold was 0.21 mA (range: 0.1–0.3 mA). A linear correlation was observed between stimulus intensity and response amplitude for intensities below 0.4 mA. Response amplitudes reached a plateau between 0.4 mA and 1.0 mA. The minimal stimulus intensity that could generate a maximal response was 0.4 mA in the orbicularis oculi (244 μV) and orbicularis oris (545 μV). In the animal study, there were no observed changes in EMG or nerve damage incidence after the continuous stimulation of 3.0 mA.

**Conclusions:**

0.4 mA is considered to be the optimal intensity of facial nerve stimulation during middle ear surgery, and it was estimated through the animal study that a stimulation of 3.0 mA is safe from facial nerve damage.

## Introduction

Facial nerve damage during middle ear surgery is a serious complication for patients and surgeons. Facial nerve damage can have severe and profound sequelae and often results in decreased self-esteem, depression, job loss, and suicidal ideation or tendencies [1]. The reported incidence of facial nerve damage during middle ear surgery is between 0.6% and 3.6% [2]. To reduce this outcome, there have been numerous attempts to develop methods that reliably identify the facial nerve during surgery. In 1965, Shedd and Durham [3] reported the first technique to identify nerves, which was intraoperative neuromonitoring (IONM). This method provides real-time identification and a functional assessment of nerves during surgery [4]. IONM has become frequently used in middle ear surgery. Since middle ear surgery is most frequently performed in patients with inflammatory disease, it is highly likely that the facial nerve will be surrounded by inflammatory soft tissue, such as granulation, and the possibility of facial nerve damage during surgery increases. Therefore, it is very important to distinguish the facial nerve from inflammatory soft tissues in the middle ear to avoid facial nerve damage.

We have routinely utilized IONM while performing middle ear surgery. IONM requires repetitive stimulation of the facial nerve during the operation, and concerns have been raised regarding the identification of a safe and optimal intensity for facial nerve stimulation during IONM to prevent facial nerve damage. Authors of previous studies on this topic have made recommendations regarding the intensity of facial nerve stimulation during middle ear surgery [5], which have varied from 0.1 mA to 1.0 mA depending on the site, state of the bony canal of the facial nerve, and the response. The “optimal” stimulus, by our definition, is the minimal stimulus intensity that can evoke the maximum electromyography (EMG) amplitude response. This stimulus intensity can help reduce the risk of nerve damage and decrease the risk of false positives or false negatives. Considering this definition, to our knowledge there has been only one report in the literature regarding the optimal intensity for stimulation of the recurrent laryngeal and vagus nerves in a prospective porcine model [6]. In addition, no prospective human studies regarding this topic on the facial nerve have been performed yet.

Therefore, the aim of this study was to prospectively investigate the optimal electrical intensity for facial nerve stimulation during middle ear surgery. We also tried to find a safe stimulation intensity through an animal study. Here, we report our findings on the EMG responses of the facial muscles, as assessed by IONM of the facial nerve.

## Materials and methods

### Patients

**Fig 1:**
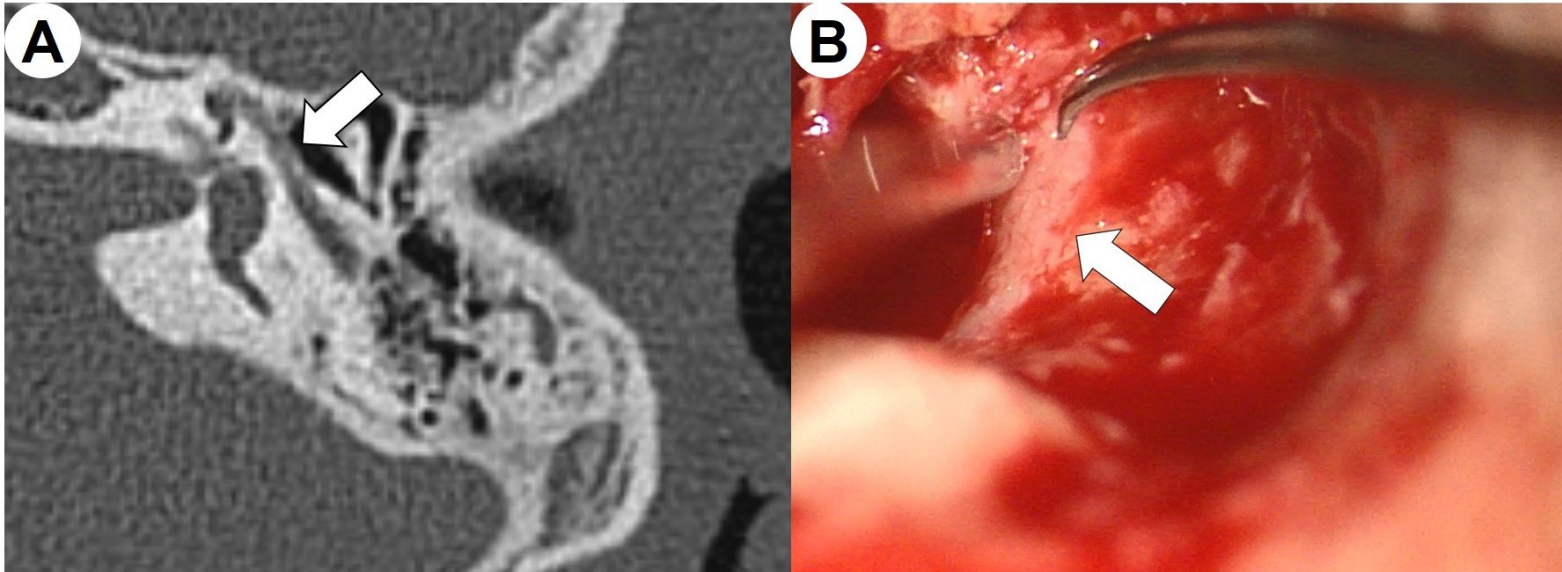
Preoperative temporal bone computed tomography scan (A) and intraoperative surgical inspection (B) showing bony canal dehiscence in the tympanic segment of the facial nerve (arrow).

Between September 2016 and September 2017, 173 patients with middle ear diseases underwent middle ear surgery in our department. Among these, there were 37 (21.4%) patients who already had their the facial nerves exposed prior to the surgery, who were subsequently enrolled in the present study. Bony canal dehiscence of the facial nerve was predicted using computed tomography scans of the temporal bone and confirmed by surgical inspection (Fig 1). This study included only patients with bony canal dehiscence of the facial nerve that did not have other pathologies. This inclusion criterion assures that incorrect and incomplete results do not occur from the intact bony canal and soft tissues being present around the facial nerve.

To implement data standardization, a single experienced surgical otologist performed all operations and identical protocols for inhalants and muscle relaxants were applied to all patients. All study patients received IONM during surgery and exposure of the facial nerve was confirmed by electrical stimulations. This prospective human study was approved by the Korea University Anam Hospital Institutional Review Board (ED17104). In addition, written informed consent was obtained from all patients.

### Intraoperative neuromonitoring

The NIM® 3.0 nerve monitoring system (Medtronic, Inc., Jacksonville, FL, USA) was used in all patients. Electrical nerve stimulations were performed on the exposed facial nerves using monopolar probes. The monopolar probes delivered the stimulus to the facial nerves at a frequency of 4 pulses/second for 100 μs. The default setting of stimulus intensity was 0.8 mA during middle ear surgery and the manufacturer’s recommended intensity for facial nerve stimulation was 0.3 mA. We utilized intensities of 0.1–1.0 mA (increased stepwise by increments of 0.1 mA) and the response amplitudes were recorded. The stimulus intensity at which an EMG response amplitude of >50 μV was first evoked was defined as the response threshold. In addition, EMG recordings were obtained from the orbicularis oculi and orbicularis oris using needle electrodes.

### Animal study

**Fig 2:**
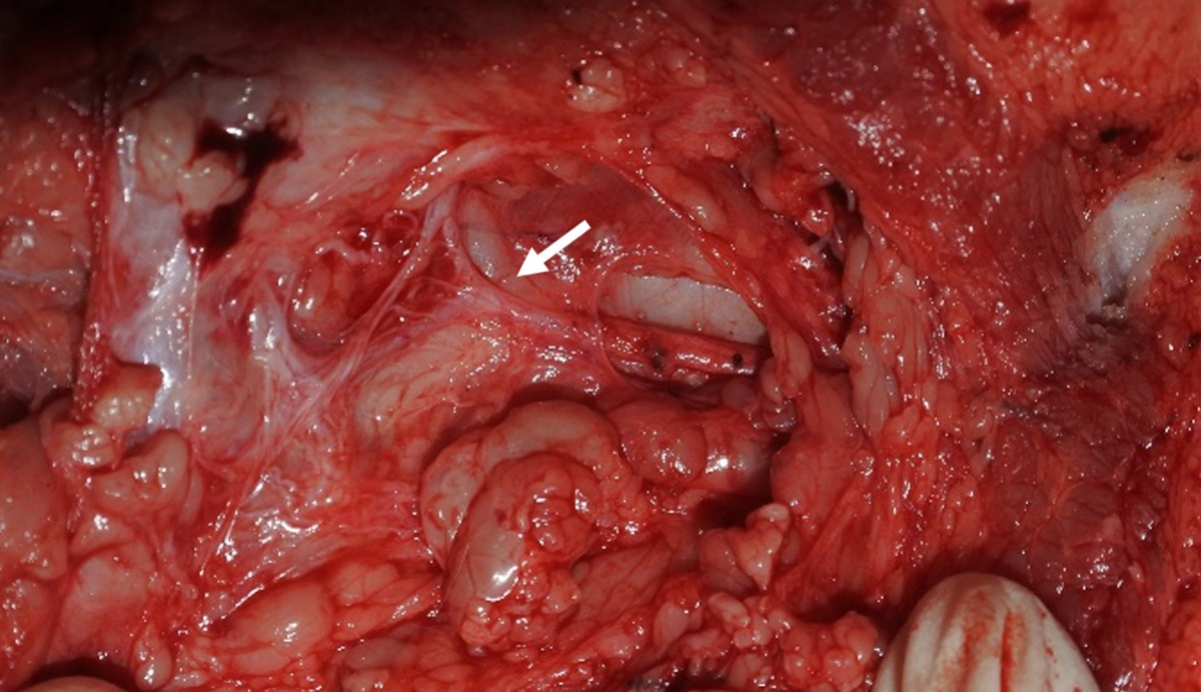
Surgical anatomy of the Landrace-Yorkshire-Duroc (LYD) pig with facial nerve (arrow).

Four female Landrace-Yorkshire-Duroc (LYD) adult pigs (XP Bio, Inc., Anseong, Korea) aged 8–10 months and weighing between 20–25 kg were enrolled in an animal study. They were kept in separate cages in a controlled environment with humidity at 50%, constant temperature at 22°C, once daily feeding with ad libitum access to water. A single experienced veterinarian anaesthetized the pigs with intravenous thiopenthal (15 mg/kg) for induction and maintained with isoflurane (1.5% to 3%). Blood pressure, heart rate, oximetry, and airway pressure, were continuously monitored. The facial nerve was identified by exposing the parotid gland with an infra-auricular incision, similar to parotidectomy procedures. The nerve was dissected away from around muscle, fat, and fascia (Fig 2). Intraoperative neuromonitoring was used with the same instruments and methods as the human studies. After confirming baseline EMG response, the facial nerve trunk was continuously stimulated for 10 minutes (3.0 mA; 4 Hz; width, 100 μs). The EMG responses before and after stimulation were compared. Then, the stimulated nerve was excised and the nerve damage was confirmed pathologically. A total of 8 nerves were studied on bilateral facial nerve of the pigs. After the procedure, the pigs were euthanized via intravenous injection of 20 ml saturated potassium chloride during anesthesia. The animal study was approved by the Institutional Animal Care and Use Committee of Korea University College of Medicine (KOREA-2017-0186).

## Results

### Demographics

Patient demographic data is shown in Table 1. Thirty-seven patients (14 men and 23 women; age = 17–62 years; mean age = 53.3 ± 13.1 years) underwent middle ear surgery in our department. Thirty-three (91.8%) of the 37 patients had dehiscence in the tympanic segment. As expected, the tympanic segment was the most common site of dehiscence. Two (5.4%) were dehiscent in the second genu portion and five (13.5%) were dehiscent in the mastoid segment. Tympano-mastoidectomy were the most common procedures in this study.

**Table 1:** Demographic data. The tympanic segment was the most common site of the facial nerve dehiscence and tympano-mastoidectomy were the most common procedures in this study.

Parameters	Result
Age, mean ± SD (year)	53.3 ± 13.1
Gender, female (n)/male (n) ratio	23/14
Dehiscent sites of the facial nerve (multiple)	
Tympanic segment, n (%)	34 (91.8)
2^nd^ genu, n (%)	2 (5.4)
Mastoid segment, n (%)	5 (13.5)
Surgical procedure	
Tympanoplasty, n (%)	6 (16.2)
Tympano-mastoidectomy, n (%)	28 (75.7)
Ossiculoplasty, n (%)	3 (8.1)

### Minimal stimulation threshold

The mean minimal facial nerve stimulation threshold was 0.21 mA (range: 0.1–0.3 mA). The stimulation threshold was defined as the stimulus intensity needed to evoke an EMG response with an amplitude of >50 μV, which was also the default threshold parameter from the manufacturer.

### EMG response significantly increased to 0.4 mA

**Fig 3:**
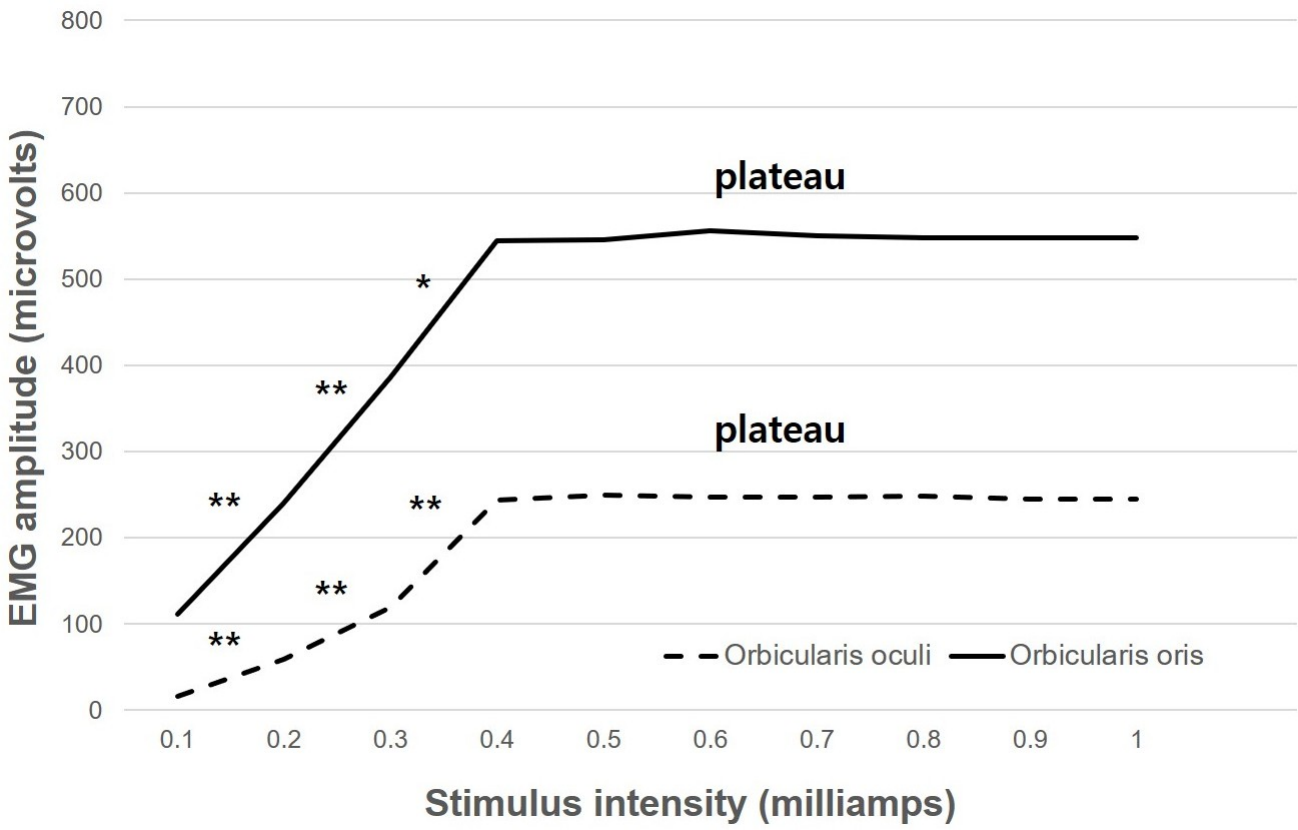
The linear correlation between the stimulus intensity and the response amplitude below 0.4 mA. The response amplitude plateaued between 0.4 mA and 1.0 mA. The plateau indicates that was no significant difference between the interstimulus intensity. The minimum intensity of the stimulus that could generate a maximum response was 0.4 mA in the orbicularis oculi (244 μV) and orbicularis oris (545 μV). *P<0.05, **P<0.01, paired *t*-test.

EMG response amplitudes at different facial nerve stimulation intensity levels are shown in Table 2 and Fig 3. A linear correlation was observed between the stimulus intensity and the response amplitude for intensities below 0.4 mA. The responses of the orbicularis oculi and orbicularis oris at an intensity of 0.1 mA were 16 μV and 111 μV, respectively. At 0.2 mA, the responses of the orbicularis oculi and orbicularis oris were 59 μV and 240 μV, respectively. Lastly, at 0.3 mA, the responses of the orbicularis oculi and orbicularis oris were 119 μV and 387 μV, respectively. The EMG amplitude significantly increased as stimulation intensity increased.

**Table 2:** EMG amplitude response from different stimulus intensities. EMG amplitude response significantly increased to 0.4 mA of stimulus intensity and plateaued within 0.4–1.0 mA of stimulus intensity.

Stimulus intensity, mA	Orbicularis oculi		P-value	Orbicularis oris		P-value
	EMG amplitude, μV			EMG amplitude, μV		
	Mean ± SD	%*		Mean ± SD	%*	
0.1	16 ± 3	6.5		111 ± 36	20.2	
			< 0.01**			<0.01**
0.2	59 ± 32	24.1		240 ± 185	43.7	
			<0.01**			<0.01**
0.3	119 ± 86	48.5		387 ± 242	70.6	
			<0.01**			0.02*
0.4	244 ± 110	99.5		545 ± 359	99.4	
			0.85			0.99
0.5	249 ± 121	101.6		546 ± 340	99.6	
			0.93			0.91
0.6	247 ± 78	100.8		556 ± 277	101.4	
			0.99			0.94
0.7	247 ± 62	100.8		550 ± 234	100.3	
			0.96			0.98
0.8	248 ± 113	101.2		548 ± 280	100.0	
			0.90			0.99
0.9	245 ± 114	100.0		548 ± 302	100.0	
			0.99			0.99
1.0	245 ± 161	100.0 (reference)		548 ± 287	100.0 (reference)	

*EMG =* electromyography, *SD* = standard deviation

The mean percentage of responses uses amplitudes from 1.0 mA stimulation as a reference.

The p-value indicates the differences in EMG response for the different stimulus intensities.

Significant results are represented by *P<0.05, and **P<0.01.

### EMG response plateaued within 0.4–1.0 mA

The response amplitude plateaued between 0.4 mA and 1.0 mA (Table 2 and Fig 3). We defined the plateau as the amplitudes where no significant differences between the inter-stimulus intensities occurred. The mean amplitudes of the orbicularis oculi and the orbicularis oris were 246.6 μV (range: 244–249 μV) and 548.8 μV (range: 545–556 μV), respectively. The minimum intensity at which the stimulus could generate a maximum response was 0.4 mA in both the orbicularis oculi (244 μV) and orbicularis oris (545 μV) (Table 2 and Fig 3).

### Postoperative results: No facial paralysis in any of the patients

Preoperative and postoperative facial nerve functions were documented according to the House-Brackmann facial nerve grading system [7]. There was no observed postoperative facial paralysis in any of the patients.

### Animal study

**Fig 4:**
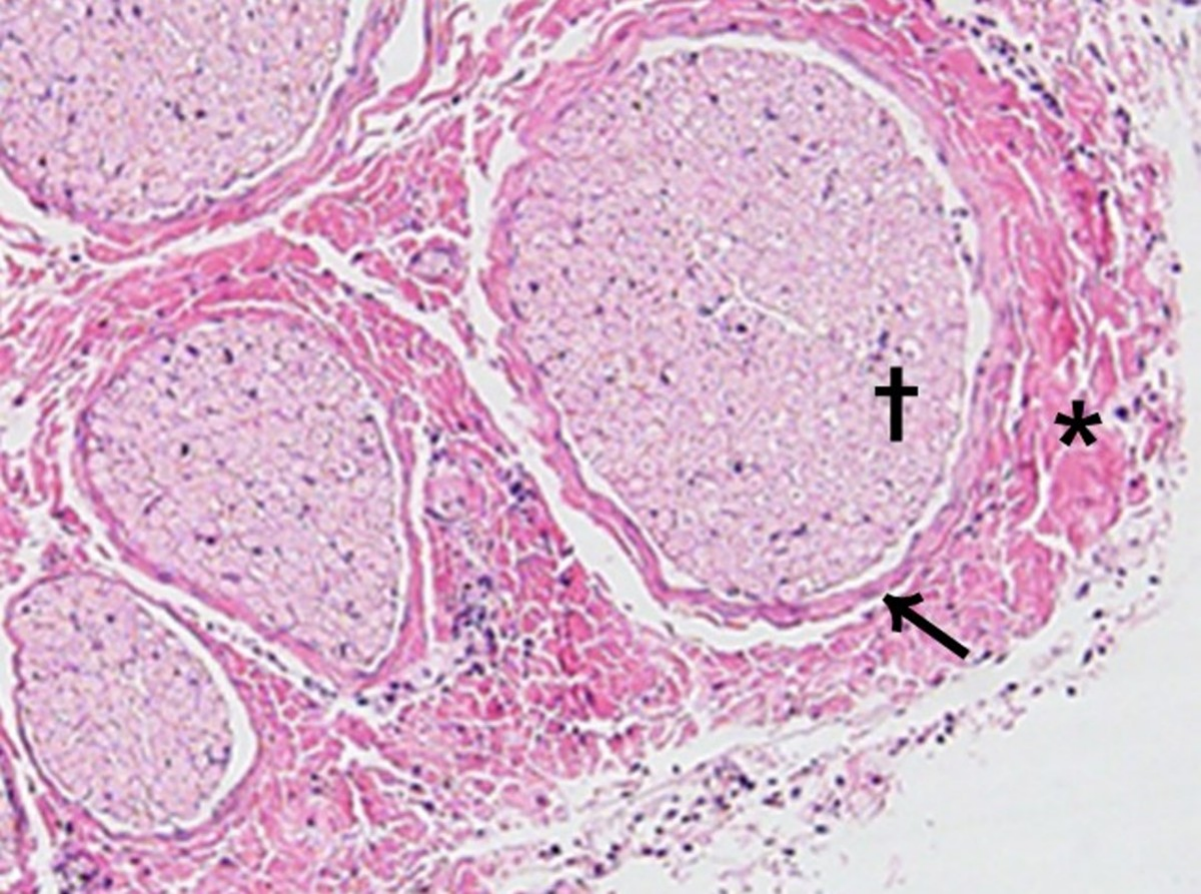
Microscopic findings of continuously stimulated facial nerve (H&E, x100). There was no evidence of necrosis and inflammation around the epi (asterisk), peri (arrow), endoneurium (dagger) of the facial nerve after 10 minutes continuous stimulation of 3.0 mA stimulus intensity.

In the animal study, when the facial nerve of the pig was stimulated at 3.0 mA, mean EMG results were 949 μV for orbicularis oculi and 1399 μV for orbicularis oris muscles. EMG results after 10 minutes of continuous stimulation showed no significant difference from before stimulation in all pigs. The stimulated nerve was then excised and examined under a microscope by an experienced pathologist. No evidence of nerve necrosis or inflammation was found (Fig 4).

## Discussion

During middle ear surgery, the facial nerve can easily be damaged because of its close proximity to the labyrinth and ossicles. Dehiscence of the facial nerve is usually mostly congenital, but may also occur as a result of inflammatory diseases or tumorous conditions, such as cholesteatoma [8], and it significantly increases the risk of iatrogenic injury. Results of the present study correspond with those from earlier studies that report the tympanic segment is the most common site for dehiscence of the facial nerve (91.8%). The proportion of tympanic segment dehiscence has varied from 85% to 90.7% [4, 9]. Therefore, the tympanic segment is the most critical site of the facial nerve and careful manipulation of the area is necessary.

The role and importance of IONM is widely recognized and accepted. In a previous study, Heman-Ackah et al. introduced the role of IONM in relation to the facial nerve for otological surgery [5]. In their study, facial nerve dehiscence was reported as 13 to 43%, and the probability of detecting a facial nerve dehiscence as IONM was reported as 89 to 100%. These results are similar to those in our study (21% and 100%, respectively). The high rates of nerve detection are assumed to be attributed to advances in technology. The summary of the predictive role of IONM in relation to the facial nerve is shown in Table 3.

**Table 3:** Summary of the predictive role of the facial nerve IONM. Facial nerve dehiscence was reported as 13 to 43%, and the probability of detecting a facial nerve dehiscence as IONM was reported as 89 to 100%.

Study (Year)	No. of Procedures	Device	Facial Nerve Dehiscence (%)	Facial Nerve Dehiscence Detected by IONM (%)
Pensak et al. (1994)	250	Xomed NIM-2	95 (38%)	88 (93%)
Noss et al. (2001)	262	In-house components	35 (13%)	31 (89%)
Choung et al. (2006)	100	Xomed NIM-2	43 (43%)	43 (100%)
Park et al. (this study)	173	Medtronics NIM 3.0	37 (21%)	37 (100%)

During surgery with IONM, stimulating currents are chosen based on the individual surgeon’s experience. Due to the variance in experiences, controversy remains regarding safe and optimal intensities that generate sufficient response amplitudes. In a previous study, Choung et al. reported that an electrical stimulation intensity of 0.7 mA for the first screening and 0.4 mA for the second screening are considered appropriate for identifying the facial nerve using IONM during middle ear surgery [10]. Furthermore, Liu et al. reported that since each study patient achieved the maximum response amplitude in their study, a stimulation intensity of 0.2 mA was optimal for the internal auditory canal (IAC) segment of the facial nerve [11]. In the current study, we suggest that 0.4 mA should be selected as the initial current for facial nerve stimulation.

IONM has recently been applied during otorhinolaryngology surgery [4]. The procedure has not only been utilized during middle ear surgery, but also in thyroid and parotid surgeries, as well as in other areas in related studies that are currently underway. Currently, continuous IONM (CIONM) is applied to the vagus nerve during thyroid surgery [12]. Although CIONM is not considered to be useful in middle ear surgery, further research may reveal some applications. For middle ear surgeries, there have been novel methods developed to perform facial nerve monitoring during the surgical procedure [13–15]. Fluorescence-assisted visualization instruments, detachable magnetic nerve stimulators, and transcutaneous stimulators are a few novel techniques that may be used in future surgeries. However, further research regarding the efficacy and safety of these new techniques is required.

One limitation of the present study is that there is no information on the extent of the maximum allowable intensity in humans. Previous studies have recommended that the intensity of facial nerve stimulation can be up to 1.0 mA. Thus, we studied stimulation intensity up to 1.0 mA. However, ethically, it is difficult to perform this important research in humans. Therefore, we tried to estimate the safest stimulus intensity for the facial nerve from the animal study. In a previous study of the vagus and recurrent laryngeal nerves in piglets, it was estimated that there was no nerve injury or deterioration in function at 3.0 mA for 10 minutes of continuous stimulation [6]. In our animal study, it was estimated that the stimulation of 3.0 mA to the facial nerve did not result in nerve damage and was therefore safe. However, it is difficult to apply the results of animal studies directly to humans, and the possibility of excessive stimulation that causes nerve damage should be considered. Therefore, facial nerve stimulation should be gradually increased from 0.4 mA as confirmed in this study, and possible excessive stimulation should be avoided.

## Conclusion

The minimum intensity of stimulus required to generate the maximum EMG amplitude, approximately 0.4 mA in this study, can be selected as the optimal intensity for facial nerve stimulation. 3.0 mA stimulation of the facial nerve is also presumed to be safe, although results of animal experiments cannot be applied directly to humans; therefore, further studies on this subject are necessary.

## Supporting information

S1 Checklist The ARRIVE guidelines checklist. (PDF)
